# Genetic Evaluation of Dual-Purpose Buffaloes (*Bubalus bubalis*) in Colombia Using Principal Component Analysis

**DOI:** 10.1371/journal.pone.0132811

**Published:** 2015-07-31

**Authors:** Divier Agudelo-Gómez, Sebastian Pineda-Sierra, Mario Fernando Cerón-Muñoz

**Affiliations:** 1 Corporación Universitaria Lasallista, Facultad de Ciencias Administrativas y Agropecuarias, Grupo de Investigación Sobre Producción, Desarrollo y Transformación Agropecuaria, Caldas-Antioquia, Colombia; 2 Universidad de Antioquia, Facultad de Ciencias Agrarias, Grupo de investigación en Genética, Mejoramiento y Modelación Animal, (GaMMA), Medellín, Colombia; CSIRO, AUSTRALIA

## Abstract

Genealogy and productive information of 48621 dual-purpose buffaloes born in Colombia between years 1996 and 2014 was used. The following traits were assessed using one-trait models: milk yield at 270 days (MY270), age at first calving (AFC), weaning weight (WW), and weights at the following ages: first year (W12), 18 months (W18), and 2 years (W24). Direct additive genetic and residual random effects were included in all the traits. Maternal permanent environmental and maternal additive genetic effects were included for WW and W12. The fixed effects were: contemporary group (for all traits), sex (for WW, W12, W18, and W24), parity (for WW, W12, and MY270). Age was included as covariate for WW, W12, W18 and W24. Principal component analysis (PCA) was conducted using the genetic values of 133 breeding males whose breeding-value reliability was higher than 50% for all the traits in order to define the number of principal components (PC) which would explain most of the variation. The highest heritabilities were for W18 and MY270, and the lowest for AFC; with 0.53, 0.23, and 0.17, respectively. The first three PCs represented 66% of the total variance. Correlation of the first PC with meat production traits was higher than 0.73, and it was -0.38 with AFC. Correlations of the second PC with maternal genetic component traits for WW and W12 were above 0.75. The third PC had 0.84 correlation with MY270. PCA is an alternative approach for analyzing traits in dual-purpose buffaloes and reduces the dimension of the traits.

## Introduction

Buffalo herds are managed under dual-purpose production systems in Colombia so farmers are interested in improving traits related with breeding, milk and meat production. A strategy to improve herd productivity is to select animals according to their breeding values (BV), which allow programming mating according to specific objectives. However, when BV are available for various traits it can be difficult to select the animals, especially when the traits have negative genetic correlation.

The principal components analysis (PCA) is a multivariate technique that reduces the amount of originally-correlated variables into a smaller set of non-correlated variables, keeping most of the original variability, and reducing the dimensionality to a new set of variables named principal components (PC), under the assumption of losing the least possible amount of information. This technique creates orthogonal axes which are linear combinations of the original variables, based on the matrix eigenvalues of the variables considered. The eigenvalues are generated in order from highest to lowest and each eigenvalue is assigned a principal component allowing each PC to retain more variability than the following PC [1]. According to Meyer K [2], when the original variables are highly correlated the first PCs can explain most of the variation, thus allowing to eliminate redundant information.

Quantitative genetics has developed three uses for principal components (PCs): as a tool to visualize genetic variation patterns, to define the genetic parameters to be estimated, and to separate the original number of variables into a smaller set of principal components to estimate the genetic parameters of these PCs [3].

The PCA technique has been incorporated into genetic evaluations in beef cattle [4–6], dairy [7], and to analyze reproductive traits in different breeds [8–10]. Recently, PCA was used for genetic evaluations of nine traits of economic interest in buffalo cattle in Brazil, concluding that four PCs are sufficient to explain the covariance structure of the traits [11]. The reviewed literature concludes, among other things, that the PCA allows lowering dimensionality of the variables, facilitating the interpretation of data in a few PC, and identifying the type of relationship between the original variables.

The aim of this study was to explore the relationship between BV for growth, milk yield, and age at first calving in dual-purpose buffaloes by using PCA.

## Materials and Methods

### Materials

This study was approved by the Ethics Committee for Animal Experimentation of Universidad de Antioquia (approved on May, 2013, 83 minutes).

The Colombian Association of Buffalo Breeders (ACB) provided the database used in this study. The traits evaluated were: weaning weight (WW), yearling weight (W12), weight at 18 months of age (W18, view S1 Dataset), weight at 2 years of age (W24), milk yield at 270 days (MY270), and age at first calving (AFC). The age range allowed for WW, W12, W18, W24 and AFC was 180 to 300, 330 to 390, 450 to 510, 680 to 760, and 760 to 1500 days, respectively. MY270 was estimated following the guidelines of the International Committee of Animal Recording (ICAR) [12]. Animals were grazing on pastures and received mineral supplementation. The breeding system consisted in controlled natural mating. Records were taken between 1996 and 2014. All herds are located in Colombia's Caribbean region in a rainforest zone (height above sea level: 80 m, temperature: 28°C, and annual precipitation: 2000 mm) [13]. All herds were managed as dual-purpose systems. The database (S2 Dataset pedigree dataset available) included a relationship matrix with 48621 animals, predominantly Murrah crossbreds. An overview of the data is shown in Table 1.

**Table 1:** Weights at weaning (WW), one year of age (W12), 18 months of age (W18), 2 years of age (W24), milk yield at 270 days (MY270), and age at first calving (AFC) for dual-purpose buffaloes in Colombia.

Trait	SX	n	Mean	CV
WW (kg)	M	12479	208.70	0.23
	F	11527	205.85	0.23
W12 (kg)	M	3045	213.27	0.21
	F	4184	208.92	0.20
W18 (kg)	M	1309	262.56	0.20
	F	2677	252.94	0.19
W24 (kg)	M	454	381.86	0.16
	F	2292	349.24	0.14
MY270 (kg)	F	15159	1044.00	0.23
AFC (days)	F	4244	1109.00	0.11

SX = sexo, (M = male, F = female), n = number of males or females, CV = coefficient of variation

### Genetic parameters

One-trait models were used for estimating genetic parameters and breeding values with MTDFREML (Multiple trait Derivate-Free Restricted Maximum Likelihood) [14].

For WW and W12 the random effects were: direct additive genetic (*a*), maternal additive genetic (*m*), maternal permanent environmental (*pe*), and residual effect (ε). The fixed effects were: sex (male or female), number of calving (1 to 14) and contemporary group (farm, year, and birth time: January to April, May to August, or September to December). Age at weighing was used as a covariate (linear effect). The matrix representation of the model is:

$$y=X\beta +Z_{1}a+Z_{2}m+Wpe+\varepsilon .\;\text{With }Cov\left(Z_{1}a,\;Z_{2}m\right)=0$$

Where *y* is a vector of observations, *β* is the vector of fixed effects, and *ε* is the random residual vector. *X*, *Z*_*1*_, *Z*_*2*_, and *W* are the incidence matrices relating the fixed effects, direct additive genetic effects, maternal additive genetic effects, and maternal permanent environmental effects, respectively.

The following formula

$$h_{t}^{2}=\left(\sigma_{a}^{2}+0.5*\sigma_{m}^{2}+\sigma_{am}\right)/\sigma_{p}^{2},$$

by Willham RL [15], was used in the estimation of total heritability for WW and W12. Where

*h* ^*2*^_*t*_ = total heritability

*σ* ^*2*^_t_ = direct additive genetic variance

*σ* ^*2*^_*m*_ = maternal additive genetic variance

*σ*_*am*_ = genetic covariance between direct and maternal effects

*σ* ^*2*^_*p*_ = phenotypic variance

For W18 and W24 random effects were the additive genetic random (*a*) and the residual effect (ε). The fixed effects were sex (male or female), number of calving (1 to14), and contemporary group (defined as for WW and W12). The age at weighing was used as a covariate (linear effect).

For AFC the random effects were the same as for W18 and W24, and the fixed effect of contemporary group was included (farm, year, and time of first birth: January to April, May to August, or September to December).

The matrix representation of the model was:

y = XB + Z1a+ Wpe + ε

For MY270 the random effects were: additive genetic (*a*), permanent environmental (*pe*), and residual effect (ε). The fixed effects were parity (1 to 14) and contemporary group (farm, year, and time of birth: January to April, May to August, or September to December).

The matrix representation of the model was:

y = XB + Z1a+ Wpe + ε

### Principal components

PCA was developed using the BV from 133 males, selected from 961 males with higher than 50% reliability for WW, W12, W18, W24, MY270, AFC, maternal genetic effect for weaning weight (MGWW), and maternal genetic effect for yearling weight (MGW12), data are also available in S3 Dataset. All BV were standardized to zero mean and unit variance. To select the number of principal components (PC) that explained the highest percentage of variance only those PC with greater than one eigenvalues were took into account [16]. The linear correlations of traits with each PC were estimated, and significant traits in each PC were defined. This analysis was conducted using command PCA, FactoMineR library [17] of r-project software [18].

## Results

### Genetic parameters

The estimated heritability of the studied traits is presented in Table 2. Traits with the highest and lowest heritability were W18 and AFC, with 0.53 and 0.17, respectively. Heritability of the other traits ranged between 0.18 and 0.23. Heritability of the maternal genetic component included in WW and W12 was 0.04 and 0.08, respectively, indicating the need to include this effect in genetic assessments to obtain more accurate heritabilities for these two traits. Heritabilities of the permanent environment for WW, W12, and MY270 were 0.11, 0.16, and 0.25, respectively.

**Table 2:** Direct heritability (h^2^_*a*_), maternal heritability (h^2^m), permanent environment (c^2^) and total heritability (h^2^_*t*_) of dual-purpose buffaloes in Colombia.

Trait	h^2^_*a*_	h^2^_m_	c^2^	h^2^_*t*_
WW	0.16	0.04	0.11	0.18
W12	0.16	0.08	0.16	0.20
W18	0.53			
W24	0.21			
MY270	0.23		0.25	
AFC	0.17			

WW: weaning weight, W12: yearling weight, W18: weight at 18 months of age, W24: weight at two years of age, MY270: milk yield at 270 days AFC: age at first calving

### Principal component analysis

PCA was performed using BV of WW, W12, W18, W24, MY270, AFC, MGWW and MGW12 from 133 breeding males chosen from 961 males. The first three PC had eigenvalues greater than one, and explained 65.78% of the original variance of the breeding values for the aforementioned traits, view Table 3. See PCA progam in S1 File.

**Table 3:** Eigenvalues and variance proportions for the principal components (PC) of the genetic values.

PC	Eigenvalue	Variance proportion	Cumulative variance proportions
**1**	2.59	0.32	0.32
**2**	1.59	0.20	0.52
**3**	1.07	0.14	0.66
**4**	0.78	0.10	0.76
**5**	0.65	0.08	0.84
**6**	0.58	0.07	0.91
**7**	0.42	0.05	0.96
**8**	0.28	0.04	100.00

**Fig 1:**
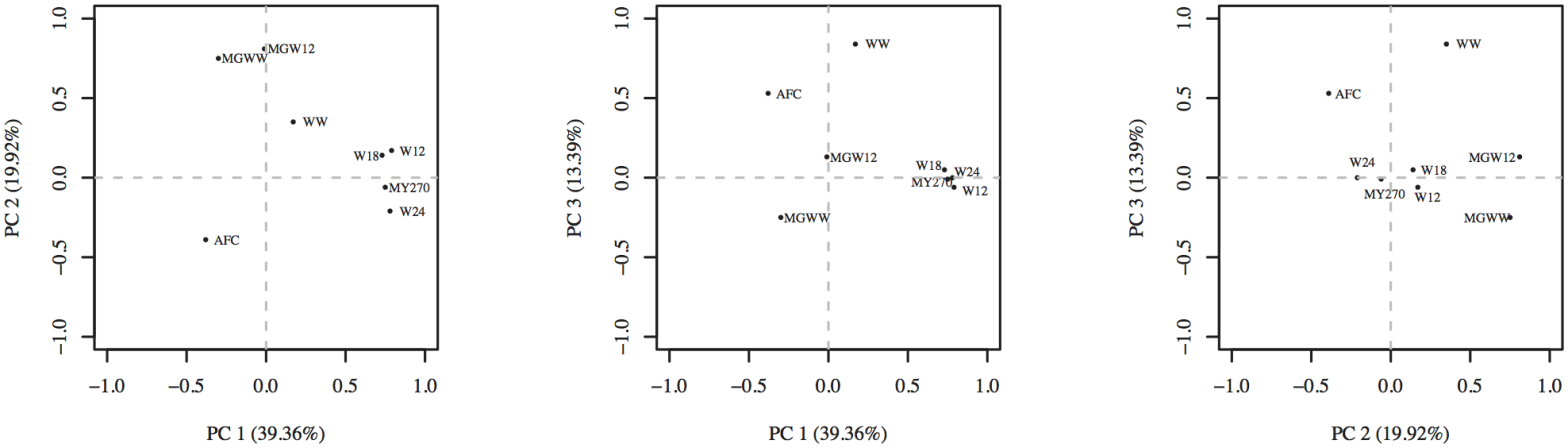
Distribution of the traits analyzed in each of the first three principal components (PC1 vs PC2, PC2 vs PC3 and PC2 vs PC3).

Distribution of traits in each of the first three components (PC1, PC2 and PC2) is shown in Fig 1. The lines represent eigenvectors indicating the strength and direction in each PC [19]. Traits WW, W12, W18 and W24 showed greatest intensity in PC1, and related positively with this component. The MY270 behaved in similar way, but with less intensity. On the other hand, AFC and MGWW were negatively associated with PC1, while MGWW, MGW12 and MY270 related positively with PC2. The traits with greatest intensity in PC3 were MY270 and AFC, and they were positively related. The MGWW was negatively associated with this component, and had low intensity (Fig 1).

Table 4 shows the correlations of significant traits with each of the first three PC. The PC1 presented higher than 0.72 correlation with WW, W12, W18, and W24; and it was -0.38 and -0.30 with AFC and MGWW, respectively. Correlation of PC2 with WW and AFC was negative, while it was positive with MY270, MGWW and MGW12. Correlation of PC3 with MY270 and AFC was positive, and it was negative with MGWW.

**Table 4:** Linear correlation of genetic values for the traits that were significant with principal components (PC1, PC2 and PC3).

Trait	PC1	PC2	PC3
**WW**	0.78	-0.21	
**W12**	0.75		
**W18**	0.78		
**W24**	0.73		
**MY270**	0.17	0.35	0.84
**AFC**	-0.38	-0.39	0.53
**MGWW**	-0.30	0.75	-0.25
**MGW12**		0.82	

PS weaning weight, W12: yearling weight, W18: weight at 18 months, W24: weight at 2 years, MY270: milk yield at 270 days AFC: age at first calving, MGWW: maternal genetic effect for weaning weight, MGW12: maternal genetic effect for weight at one year of age

## Discussion

The values found in this study for WW and W12 were higher, and W18 and W24 were lower than those reported in Colombia for those traits: 182, 201, 278 and 363 kg, respectively [20]. Milk yield was lower to 2286.8 kg reported for buffaloes in Italy [21], and 1594 kg reported for Murrah buffaloes in Brazil [22]. AFC was higher to 1094 days reported for Murrah buffaloes in Brazil [22] and less than 1140 days reported for buffaloes in Colombia [23].

The performance parameters of buffaloes for WW, W12, W18, W24, MY270, and AFC were better than the data reported for the dual purpose cattle in Colombia [24], indicating that buffalo is a good livestock production alternative in this country.

The estimated WW, W12 and W24 heritability was lower than the figures reported in Colombia by Bolivar et al. [23]: 0.42, 0.42 and 0.41, respectively. Heritability of W18 was 0.42 in that report, which is lower than estimated in the present study.

In this study, the estimated heritability for milk yield was lower than previously reported for buffaloes in Brazil: 0.30, 0.25, and 0.28 [11,25,26], respectively, but was higher than that reported in Italy 0.14 [21], Brazil 0.22 [27] and Colombia 0.22 [28].

The estimated heritability for AFC was higher than that reported in Nellore heifers: between 0.08 and 0.16 [29]; but less than 0.47 estimated in buffaloes in Colombia [23].

The estimated maternal heritability for WW and W12 coincide with values reported by Albuquerque and Meyer [30] for Nellore cattle. They evaluated this trait from birth to 600 days of age, reporting values between 0.01 and 0.08 that were statistically significant at up to 390 days. In Brazil Malhado et al. [31] estimated maternal heritability as 0.09 for weight at 205 days of age in buffaloes. In Colombia Bolivar et al. [20] reported 0.28 for the same trait for weaning weight. These results suggest that inclusion of the maternal effect allows for a better estimation of heritability for WW and W12. In Table 5 shows the heritability estimates for the studied traits and those obtained by other researchers.

**Table 5:** Heritabilities (h^2^) estimated for dual-purpose buffalo cattle in this study compared to other studies.

Trait	h^2^, this study	h^2^, other studies	Literature
WW	0.18	0.45	[20]
W12	0.20	0.42	[20]
W18	0.53	0.42	[20]
W24	0.21	0.41	[20]
MY270	0.23	0.25	[25]
	0.23	0.20	[26]
	0.23	0.30	[27]
	0.23	0.30	[11]
	0.23	0.14	[21]
	0.23	0.22	[28]
AFC	0.17	0.16	[29]
	0.17	0.47	[23]
MGWW	0.04	0.09	[31]
	0.04	0.01*	[30]
MGW12	0.08	0.08*	[30]

WW weaning weight, W12: yearling weight, W18: weight at 18 months, W24: weight at 2 years, MY270: milk yield at 270 days AFC: age at first calving, MGWW: maternal genetic effect for weaning weight, MGW12: maternal genetic effect for weight at one year of age.

*Ganado nelore Nelore cattle

The PCA results in this study are consistent with other reports, evidencing the usefulness of PCA to reduce dimensionality. According to the report by Val and Ferraudo [8], the first two PCs comprised 70.33% of the total variation of six traits associated with meat production and one trait associated to breeding in Nellore cattle. Also in Nellore cattle, three PCs accounted for 100% of the additive genetic variance of nine traits associated with meat production [5]. Oliveira et al. [11] evaluated seven productive and two reproductive traits of buffaloes in Brazil concluding that a reduced rank model with 3 or 4 PCs was sufficient to explain the largest percentage of the additive genetic variance for all the traits.

## Conclusions

According to the heritability figures obtained, W18 and MY270 would be the most responsive traits to the selection process, while AFC would be less responsive. PCA facilitates and improves efficiency of the animal selection process by using correlations between traits and components, hence reducing the range of the analysis. It is concluded that the traits studied in this work can be analyzed with the first three PCs.

## Supporting Information

S1 Dataset This file contains the information productive weight at 18 months W18, information of each of the columns corresponds to: animal (id), father (sire), mother (dam), sex (sx), contemporani group (cg), calving number (N), weight (W18), and age (age). (XLSX)

S2 Dataset This archive contains the genealogical information of animals tested, each of the three columns correspond to the renumbering of the animal, father and mother, respectively. (XLS)

S3 Dataset This archive contains breeding values (BVs) from 133 males used for the principal component analysis (PCA). Information of each column corresponds to BV for: milk yield at 270 days (MY270), weaning weight (WW), weight at one year (W12), weight at 18 months (W18), weight at two years (W24), age at first calving (AFC), maternal genetic effect for weaning weight (MGWW), and maternal genetic effect for yearling weight (MGW12).
(TXT)

S1 File This file contains the program for principal components analysis (PCA) in r-project. (DOCX)
