# Correction: Genetic Evaluation of Dual-Purpose Buffaloes (*Bubalus bubalis*) in Colombia Using Principal Component Analysis

**DOI:** 10.1371/journal.pone.0137148

**Published:** 2015-08-27

**Authors:** Divier Agudelo-Gómez, Sebastian Pineda-Sierra, Mario Fernando Cerón-Muñoz

There is an error in Fig 1 Please see the corrected Fig 1 here.

**Fig 1:**
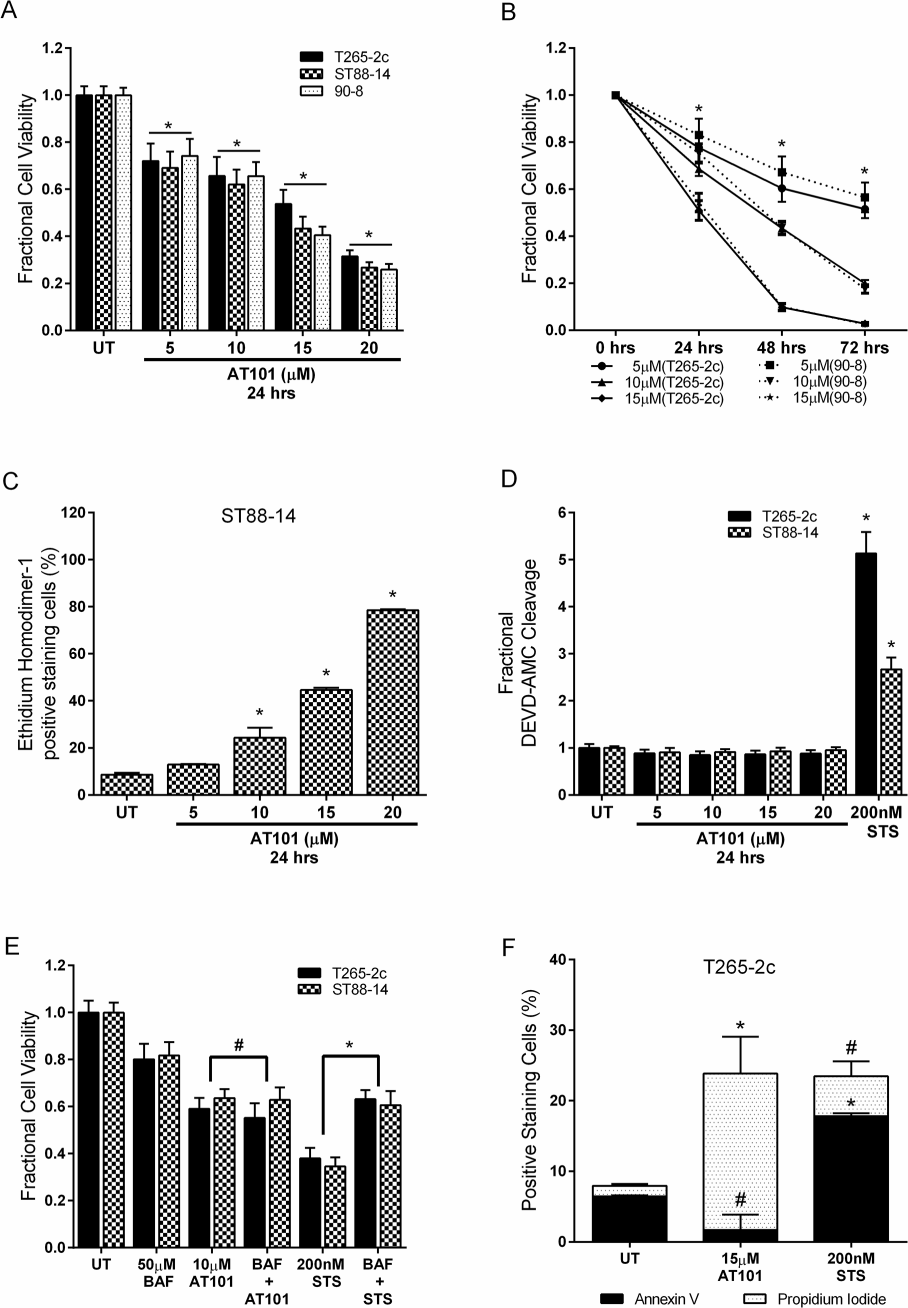
Distribution of the traits analyzed in each of the first three principal components (PC1 vs PC2, PC2 vs PC3 and PC2 vs PC3).
