# Correction: Correction: Genetic Evaluation of Dual-Purpose Buffaloes (*Bubalus bubalis*) in Colombia Using Principal Component Analysis

**DOI:** 10.1371/journal.pone.0138316

**Published:** 2015-09-14

**Authors:** The PLOS ONE Staff

There is an error in the correction published on August 27, 2015. The image of Fig 1 in the correction published on August 27, 2015, is incorrect. The publisher apologizes for the error. Please view the correct Fig 1 below.

**Fig 1:**
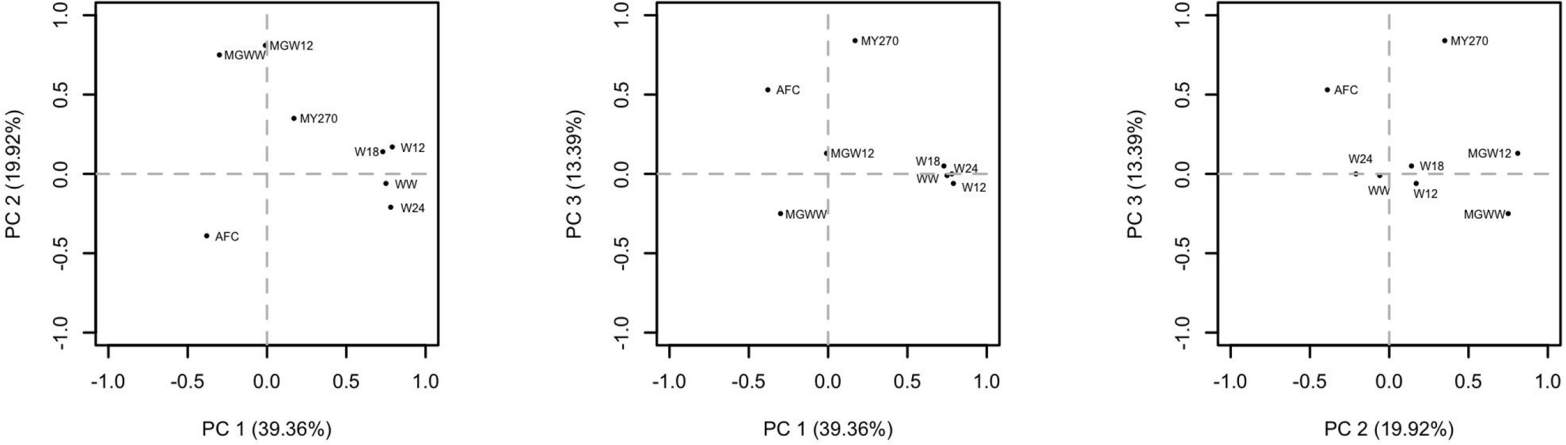
Distribution of the traits analyzed in each of the first three principal components (PC1 vs PC2, PC2 vs PC3 and PC2 vs PC3).
