# Supplementary material for: Genetic Evaluation of Dual-Purpose Buffaloes (Bubalus bubalis) in Colombia Using Principal Component Analysis
Source: PLoS One. 2015 Jul 31;10(7):e0132811. doi: 10.1371/journal.pone.0132811 (PMC4521921; doi:10.1371/journal.pone.0132811)
Supplement: S1 File — (DOCX) [file pone.0132811.s004.docx]

##Activate FactoMineR library required for the Principal Components Analysis (PCA) ##

library(FactoMineR)

# Place the PCA_Male.txt file in the desired folder ##

## Import the breeding values data set ##

S3_Dataset = read.delim("/Users/ S3_Dataset_PCA_Male.txt", header = TRUE, sep = "\t", quote = "\"", dec=",")

## Perform Principal Component Analysis(PCA) ##

res.pca <- PCA(S3_Dataset,scale.unit = TRUE)

# Obtain the general PCA summary #

summary(res.pca)

## Identify the eigenvalues, percentage of variance, and cumulative percentage of variance for each component ##

res.pca$eig

##Identify the  trait coordinates for each Principal Component (PCA)##

res.pca$var$coord
